# Supplementary material for: Tightly coupled inhibitory and excitatory functional networks in the developing primary visual cortex
Source: eLife. 2021 Dec 8;10:e72456. doi: 10.7554/eLife.72456 (PMC8654369; doi:10.7554/eLife.72456)
Supplement: Figure 2—source data 1. [file elife-72456-fig2-data1.pdf]

Figure 2 — Table 1. GABAergic signaling has net inhibitory effect on cortical activity by P21.

Animal ID	Age (Postnatal)	# events		Event frequency		Event Amplitude	
		Baseline	BMI	Baseline (Hz) (median (IQR))	BMI (Hz)	Baseline (dF/F)	BMI (dF/F)
F0232	21	25	84	0.042 (0.021 - 0.067)	0.158 (0.088 - 0.192)	0.688 (0.610 - 0.842)	0.898 (0.709 - 1.346)
F0233	21	22	100	0.033 (0.017 - 0.063)	0.175 (0.117 - 0.2)	0.638 (0.577 - 0.734)	0.784 (0.592 - 0.985)
F0089	23	149	273	0.033 (0.033 - 0.067)	0.217 (0.146 - 0.304)	0.203 (0.140 - 0.413)	0.871 (0.427 - 1.671)
F0109	21	78	275	0.016 (0.0 - 0.033)	0.233 (0.158 - 0.317)	0.279 (0.221 - 0.375)	0.618 (0.353 - 1.009)
Pooled		274	732	0.033 (0.016-0.05)	0.2 (0.129-0.271)	0.374 (0.190-0.535)	0.974 (0.470-1.220)
				p-value	*1.52E-23	p-value	*1.40E-52

Data are presented as medians (inter-quartile range). Wilcoxon rank-sum test, * p<0.001
